# Supplementary material for: Evolutionary dynamics of pro-inflammatory caspases in primates and rodents
Source: bioRxiv. 2024 Aug 27:2024.06.19.599744. Preprint. [Version 2] doi: 10.1101/2024.06.19.599744 (PMC11383037; doi:10.1101/2024.06.19.599744)
Supplement: Supplement 4 — Supplemental Data 1. Alignments and full results of GARD, CODEML, and FUBAR analyses. We provide the alignments and raw data to maximize reproducibility and data transparency. [file media-4.zip › SupData1/SupData1_README.rtf]

File description:1) The figure_alignments_trees folder contains the alignments and tree files used to create Figures 1 and 32) The annotated_loci folder contains the extracted annotated caspase loci of primates and rodents, including exon annotations for each gene. The individual files are in Genbank format and include both sequence and annotation information.3) The selection_analyses folder contains the alignment inputs and raw output files for each of our analyses of selection for each caspase gene.Within selection_analyses, each gene folder contains:1. Species tree based PAML analysis	Alignment segments based on the GARD breakpoints	PAML output files		a. NS = models 0/1/2/7/8		b. Model 8a		c. Model 0a		d. Varying codonFreq and starting omega2. Segment tree based PAML analysis	Alignment segments based on the GARD breakpoints	PAML output files		a. NS = models 0/1/2/7/8		b. Model 8a		c. Model 0a		d. Varying codonFreq and starting omega3. Datamonkey analyses	GARD .json output of breakpoints	FUBAR .csv results of each alignment segment
